# Microparticles in patients undergoing transcatheter aortic valve implantation (TAVI)

**DOI:** 10.1007/s00380-016-0885-z

**Published:** 2016-08-03

**Authors:** Christian Jung, Michael Lichtenauer, Hans-Reiner Figulla, Bernhard Wernly, Bjoern Goebel, Martin Foerster, Christoph Edlinger, Alexander Lauten

**Affiliations:** 1 Division of Cardiology, Pulmonology, and Vascular Medicine, Medical Faculty, University Hospital Düsseldorf, University Duesseldorf, Moorenstrasse 5, 40225 Düsseldorf, Germany; 2 Department of Cardiology, Clinic of Internal Medicine II, Paracelsus Medical University of Salzburg, Salzburg, Austria; 3 Department of Cardiology, Clinic of Internal Medicine I, Universitätsherzzentrum Thüringen, Friedrich Schiller University Jena, Jena, Germany; 4 Department of Cardiology, Charité-Universitaetsmedizin Berlin, Berlin, Germany

**Keywords:** Endothelial microparticles, Platelet microparticles, Endothelial dysfunction, TAVI, Aortic stenosis

## Abstract

Degenerative aortic stenosis (AS) is the most frequent form of acquired valvular heart disease. AS is known to entail endothelial dysfunction caused by increased mechanical shear stress leading to elevated circulatory levels of microparticles. Endothelial and platelet microparticles (EMP and PMP) are small vesicles that originate from activated cells and thrombocytes. We sought to evaluate whether transcatheter aortic valve implantation (TAVI) procedure would elicit effects on circulating EMP and PMP. 92 patients undergoing TAVI procedure for severe AS were included in this study. Samples were obtained at each visit before TAVI, 1 week post-procedure and at 1, 3 and after 6 months after TAVI and were evaluated using flow cytometry. A 12 month clinical follow-up was also performed. CD62E+ EMP concentration before TAVI was 21.11 % (±6.6 % SD) and declined to 20.99 % (±6.8 % SD) after 1 week, to 16.63 % (±5.4 % SD, *p* < 0.0001) after 1 month, to 17.08 % (±4.6 % SD, *p* < 0.0001) after 3 months and to 15.94 % (±5.4 % SD, *p* < 0.0001) after 6 months. CD31+/CD42b−, CD31+/Annexin+/− EMP remained unchanged. CD31+/CD41b+ PMP evidenced a slight, but statistically significant increase after TAVI and remained elevated during the entire follow-up. Apart from a procedure-related improvement in echocardiographic parameters, TAVI procedure led also to a decline in CD62E+ EMP. The reduction in pressure gradients with less hemodynamic shear stress seems also to have beneficially affected endothelial homeostasis.

**Electronic supplementary material:**

The online version of this article (doi:10.1007/s00380-016-0885-z) contains supplementary material, which is available to authorized users.

## Introduction

Degenerative aortic stenosis (AS) is the most frequent form of acquired valvular heart disease worldwide and its prevalence is expected to further increase within the aging population. Although the majority of patients have only mild valve disease, about 5 % develop severe AS [1–3]. Morbidity and mortality rates are considerably high among patients not undergoing valve replacement. Once symptoms occur in these patients, the mortality rate is approximately 50 % over the next 3 years [4].

Pathophysiology of aortic stenosis can be characterized by multiple steps: inflammation, fibrosis and finally calcification. It is not completely understood whether inflammation in aortic stenosis is a response to tissue injury or if aortic stenosis might be primarily an inflammatory disease. From the literature we know that pro-inflammatory markers such as Tumor necrosis factor-alpha (TNF-alpha), receptor activator of nuclear factor-kappa B (NF-κB) ligand (RANKL) and Interleukin-6 (IL-6) are associated with aortic stenosis [5–8]. Also elevated plasma levels of low-density lipoprotein cholesterol were associated with an augmented aortic valve calcium deposition and a higher incidence of aortic stenosis [9].

As there is no medical treatment for aortic stenosis besides vasodilators and diuretics for acute decompensation, the mainstay of treatment for aortic stenosis is still surgical or interventional aortic valve replacement [6, 10]. Ongoing inflammation in the circulatory system and increased shear stress has also been associated with endothelial dysfunction [11, 12]. Over the last few years, microparticles have come more and more into the focus of clinical scientists as they can serve as parameters to determine and monitor vascular dysfunction in several disease conditions [12–16].

Microparticles are defined as small phospholipid vesicles without a nucleus that are released from different cells, platelets, erythrocytes, leukocytes and endothelial cells [17–19]. Endothelial cell microparticles (EMP) can be identified by antigens expressed by mature endothelial cells such as CD31 [20]. EMP concentrations are known to reflect endothelial dysfunction as there are strong associations between increased circulating EMPs and both structural and functional abnormalities [21, 22]. Microparticles derived from platelets (PMP) are identified by the expression of platelet markers such as CD41 or CD61 [23]. High levels of PMPs are known to be strongly associated with thrombotic complications [24]. Horn et al. have recently shown that EMPs decrease 3 months after transcatheter aortic valve implantation (TAVI), reflecting improved endothelial function and wall shear stress [25].

The main focus of our study was to further extend our knowledge about microparticles in AS and patients undergoing TAVI. We sought to evaluate whether TAVI procedure could lead to an alteration or possible decrease in circulating levels of both endothelial and platelet microparticles epitomizing an improvement in vascular integrity and function after 1, 3 and 6 months indicating a possible long-term beneficial effect of interventional aortic valve replacement on overall endothelial function.

## Methods

The study protocol was approved by the Ethics Committee of the Friedrich-Schiller-University, Jena, Germany. The study was conducted according to the principles of the Declaration of Helsinki and Good Clinical Practice and informed consent was obtained from all patients before being enrolled.

### Study protocol

A total of 92 patients suffering from symptomatic severe AS admitted to our clinic for TAVI were enrolled in this study between November 2011 and February 2014. The diagnosis of severe AS was defined according to the guidelines of the European Society of Cardiology. Mean and peak pressure gradients (MPG and PPG), maximum velocity (*V*_max_) and aortic valve area were assessed by transthoracic echocardiography by experienced sonographers in our echo lab. All patients underwent pre-interventional screening with ECG, laboratory parameters, transthoracic and transesophageal echocardiography and coronary angiography. The obtained results were discussed in the interdisciplinary heart team. Patients that were considered not eligible for operative aortic valve replacement were planned for TAVI procedure.

TAVI procedure was performed via transfemoral access in 77 patients and via a transapical approach in 15 patients. In patients undergoing transfemoral TAVI, a 14–21 French delivery sheath was inserted in the femoral artery, depending on the size and type of TAVI-device. In patients without a permanent pacemaker, a temporary transcutaneous pacemaker was placed via transjugular access for periprocedural rapid pacing. A balloon valvuloplasty was performed during rapid pacing. Subsequently, implantation of the valve prosthesis (Edwards Sapien XT, *n* = 64; Corevalve, *n* = 11; JenaValve, *n* = 13; SJM Portico, *n* = 4) was performed. Using a closure device (Proglide; Abbott USA) the artery access was closed. For the transapical approach, an anterolateral mini-thoracotomy was performed to obtain optimal access to the apex of the left ventricle followed by pericardiotomy. The left ventricular apex was punctured with a standard access needle and an access wire was inserted. Then, a 12 French sheath was placed in the apex for ventricular access. Next, a balloon valvuloplasty was performed during rapid pacing. The valve was then introduced over the sheath and placed in the right position under angiographic control. The pharmacological regimen after TAVI in our clinic comprised of 100 mg of acetylsalicylic acid and 75 mg of clopidogrel for 3 months which was followed by a monotherapy of 100 mg of acetylsalicylic acid.

Plasma samples were obtained from all enrolled patients on the day of the procedure and 1 week post-procedure and at each of the follow-up visits after 1, 3 and 6 months.

### Flow cytometry

Microparticles in the collected blood samples were stained with antibodies against CD31, CD42b, CD62E and Annexin to analyze circulatory levels of CD31+/Annexin+, CD31+/Annexin−, microparticles using flow cytometry. EMP were defined as CD31+/CD42b− and PMP as CD31+/CD42b+.

According to previous studies [13, 18, 26], samples were centrifuged for 10 min at 2000*g*. For the preparation of platelet-poor plasma, the plasmatic supernatant was obtained without disturbing the buffy coat. Fifty microliters of platelet-poor plasma was incubated for 20 min with labeled antibodies: 4 µl of anti-CD31-PE (BD Pharmigen, USA), anti-CD62-FITC (Ancell Corporation, USA), anti-CD42b-Cy5 (BD Pharmigen, USA) and anti-Annexin-V-APC (BD Pharmigen, USA) or matching isotype controls (all from BD Pharmigen, USA). In the next step, samples were then diluted with 1 ml of phosphate-buffered saline (PBS) and analyzed on a flow cytometer (FACS Calibur, BD Biosciences, USA).

To determine the size of the acquired events, size calibration beads were added to the samples (Molecular Probes, Invitrogen, USA). MP were defined as particles that were smaller than 1.5 µm. MP were expressed as percentage of events in the relevant gate. The obtained data were processed using CellQuest Pro software (BD Biosciences, USA).

### Statistical analysis

Statistical analysis was performed using GraphPad Prism software (GraphPad Software, La Jolla, USA). All data are given as mean ± standard deviation (SD) or median with interquartile range (IQR). The Wilcoxon’s matched pairs and the paired *t*-test were utilized to calculate significances between the groups. *p* values <0.05 were considered statistically significant. Bonferroni-Holm correction was used to adjust *p* values for multiple testing. * indicates a *p* of <0.05, ** a *p* of <0.01 and *** a *p* of <0.001.

## Results

**Fig. 1:**
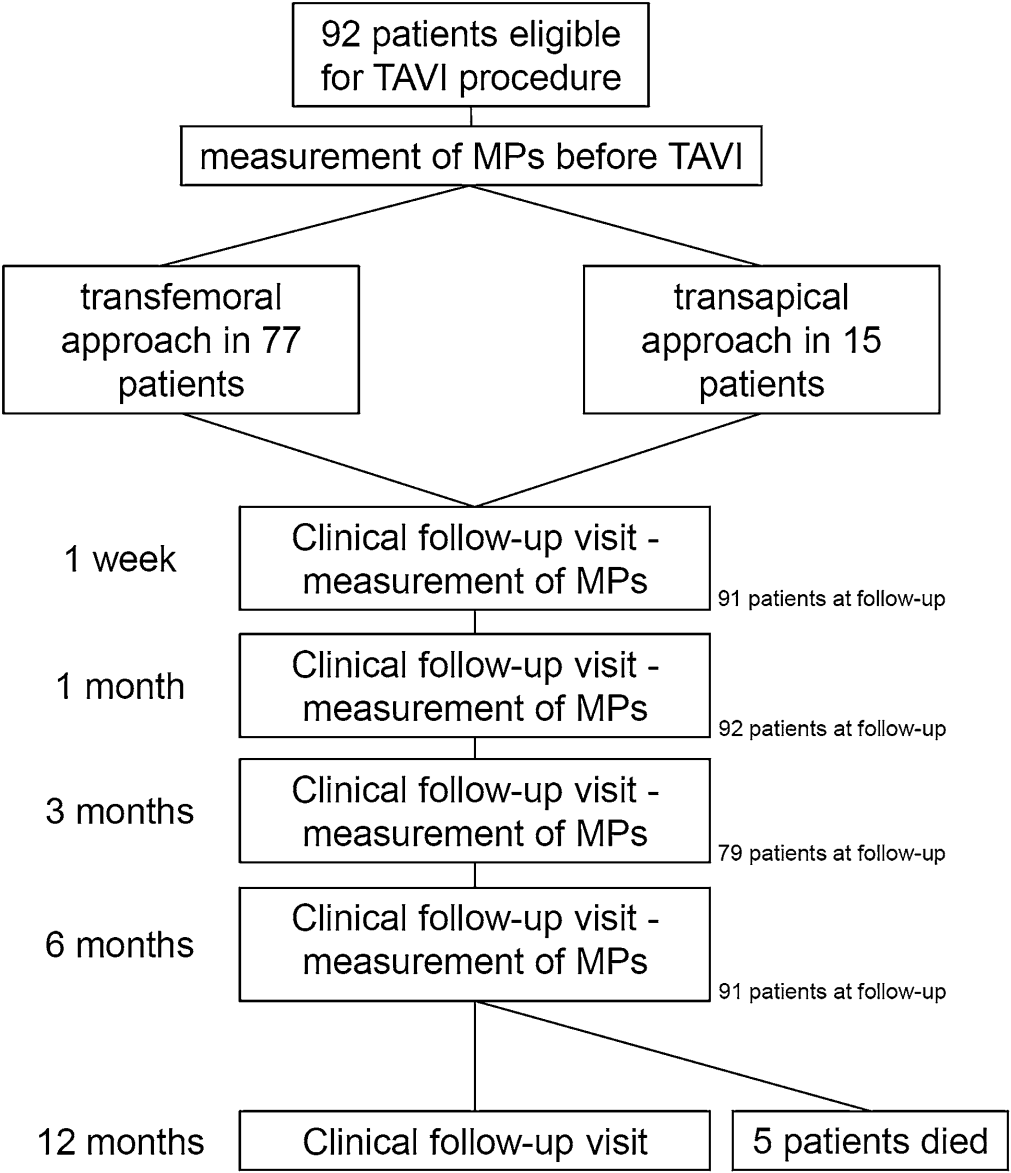
Flow chart of the study protocol

The characteristics of the study population are presented in Table 1. 92 patients with a mean age of 80.0 ± 7.13 years were included in the study and were followed-up over a period of 6 month after TAVI with periodical blood withdrawals for EMP and PMP analysis. At 12 month post-TAVI, a clinical follow-up was performed. Four different valve types were used (Edwards Sapien XT *n* = 64, Corevalve *n* = 11, JenaValve *n* = 13, SJM Portico *n* = 4). Follow-up time points with measurement of microparticles were prior to TAVI and 1 week post-TAVI and at 1, 3 and 6 months after TAVI (see Fig. 1). Mean EuroScore was 23.1 ± 14.5. TAVI procedure was performed via transfemoral access in 77 patients, whereas in 15 patients the valve was implanted via a transapical approach. None of the patients died within the 30 days after TAVI, though, five patients died within the 12 months follow-up period (mortality rate 5.4 %). Complication rates defined using VARC-2 criteria are also stated in Table 1 [27]. In total, six patients evidenced neurological deficit after TAVI (stroke *n* = 3, TIA *n* = 3). Acute kidney injury according to VARC-2 criteria occurred in two patients (stage 1 *n* = 1, stage 2 *n* = 1). Minor bleeding complications (vascular site hematoma, transfusion of <2 red blood cell concentrates) according to VARC-2 criteria occurred in 16 patients (17.4 %), major bleeding complications (transfusion of ≥2 red blood cell concentrates) in 24 patients (26.1 %) and relevant vascular access-related complications (dissection, pseudoaneurysm) in 8 patients (8.7 %). Implantation of a permanent pacemaker was necessary within 72 h in 5 patients (5.4 %). Permanent pacemaker implantation was performed in 13 % of patients after TAVI procedure, these data are comparable with previously published results [28]. Conversion to open surgery due valve malpositioning was necessary in 1 patient. Forty patients (43.5 %) were free of post-procedural aortic insufficiency, grade 0–1 (trace) aortic insufficiency was present in 21 patients (22.8 %), grade 1 in 29 patients (31.5 %) and ≥grade 2 only in 1 patient (1.1 %). Clinical and functional results after TAVI procedure were good as shown by significantly reduced *V*_max_, MPG and PPG (*p* < 0.001). Also a slight improvement in ejection fraction was documented during follow-up, from 55.9 % (±18.4 SD) before TAVI to 62.6 % (±14.3 SD) after 6 months (*p* < 0.01). A procedure-related drop in hemoglobin levels from 7.6 mmol/l (±1.1 SD) to 6.69 (±1.0 SD, *p* < 0.001) and an increase of CRP from 13.9 mg/l (±29.3 SD) to 42.5 mg/l (±29.8, *p* < 0.001) was also over served. However, at later visits levels returned to those seen before TAVI procedure. It is of note that creatinine levels did not increase significantly immediately following TAVI procedure, however, during the follow-up a rise in creatinine was found (see Table 2, *p* < 0.01).

**Table 1:** Patient characteristics of the study cohort before TAVI procedure

Demographic parameters	SD
*n*	92
Age (years)	80.0 ± 7.13
Height (cm)	163.4 ± 9.3
Weight (kg)	76.9 ± 13.3
NYHA stage	2.75 ± 1.38
Euroscore	23.1 ± 14.5

	Total (%)
Diabetes	42 (45.6 %)
Arterial hypertension	82 (89.1 %)
Coronary artery disease	68 (73.9 %)
One vessel disease	21 (22.8 %)
Two vessel disease	9 (9.8 %)
Three vessel disease	19 (20.6 %)
Myocardial infarction	12 (13.0 %)
Atrial fibrillation	5 (5.4 %)
Stroke	9 (9.8 %)
Peripheral artery disease	20 (21.7 %)
COPD	20 (21.7 %)

Laboratory parameters	Median (IQR)
Creatinine (µmol/l)	95.5 (74.0–123.8)
GFR (ml/min)	59.2 (41.4–60.0)
C-reactive protein (mg/l)	4.9 (2.0–11.1)
BNP (pg/ml)	340.5 (180.3–814.0)
Hemoglobin (mmol/l)	7.5 (7.0–8.2)
Hematokrit	0.36 (0.34–0.40)
Troponin I (ng/ml)	0.04 (0.028–0.11)
Creatinine kinase (U/l)	1.25 (0.98–1.94)

Valve parameters	Total
Transfemoral access	77 (83.7 %)
Transapical access	15 (16.3 %)
Edwards Sapien XT	64 (69.6 %)
Corevalve	11 (11.9 %)
JenaValve	13 (14.2 %)
SJM Portico	4 (4.3 %)
Valve size (mm)	26.3 ± 2.24

Complications	Total
Stroke	3 (3.3 %)
TIA	3 (3.3 %)
Acute kidney injury	
Stage 1	1 (1.1 %)
Stage 2	1 (1.1 %)
Stage 3	0 (0.0 %)

Vascular comlications	Total
Minor bleeding	16 (17.4 %)
Major bleeding	24 (26.1 %)
Life-threatening bleeding	0 (0.0 %)
Access-related complications	8 (8.7 %)
Patients requiring transfusions	28 (30.4 %)
Mean number of tranfusions	2.6
New pacemaker requirement (72 h)	5 (5.4 %)
New pacemaker requirement (7d)	12 (13.0 %)
Conversion to open surgery	1 (1.1 %)
Valve malpositioning	1 (1.1 %)
Post-procedural aortic insufficiency	
None	40 (43.5 %)
Grade 0–1 (trace)	21 (22.8 %)
Grade 1	29 (31.5 %)
≥Grade 2	1 (1.1 %)
ICU treatment (h)	26.0 (23.0–47.8)

**Table 2:** Echocardiographic and laboratory parameter before TAVI and after 1 week and at 1, 3 and 6 months of follow-up

	Pre TAVI	1 week	1 month	3 months	6 months
Ejection fraction (%)	55.9 ± 18.4	60.8 ± 12.9*	58.4 ± 16.3	63.2 ± 15.2**	62.6 ± 14.3**
*V*_max_ (m/sec)	4.34 ± 0.7	2.21 ± 0.3***	2.22 ± 0.5***	2.17 ± 0.5***	2.23 ± 0.6***
Mean pressure gradient (mmHg)	46.5 ± 14.8	11.3 ± 6.5***	11.1 ± 5.4***	11.1 ± 5.7***	11.6 ± 6.1***
Peak pressure gradient (mmHg)	77.5 ± 22.7	20.9 ± 11.3***	20.7 ± 10.8***	20.4 ± 9.8***	21.2 ± 11.1***
Aortic valve area (cm^2^)	0.66 ± 0.18	n.a.	n.a.	n.a.	n.a.
LVEDD (mm)	48.9 ± 7.4	49.8 ± 7.3	49.5 ± 8.2	47.9 ± 6.9	48.3 ± 7.5
LVESD (mm)	31.7 ± 9.4	n.a.	n.a.	n.a.	n.a.
Creatinin (µmol/l)	95.5 (74.0–123.8)	81.5 (67.5–118.8)	93.0 (75.0 to ±128.0)	101.0 (84.0–128.5)	104.5 (81.5–135.3)**
C-reactive protein (mg/l)	4.8 (2.0–11.1)	37.4 (20.0–59.6)***	4.9 (2.2 to 15.0)	3.4 (2.0–7.9)	3.1 (2.0–5.9)
hemoglobin (mmol/l)	7.5 (7.0–8.2)	6.6 (6.2–7.2)***	7.3 (6.6 to 8.0)***	7.6 (6.9–8.5)	7.9 (7.2–8.5)

Significances are expressed vs. baseline parameters before TAVI

* Indicates a *p* of <0.05, ** a *p* of <0.01 and *** a p of <0.001

*n.a* data not available

**Fig. 2:**
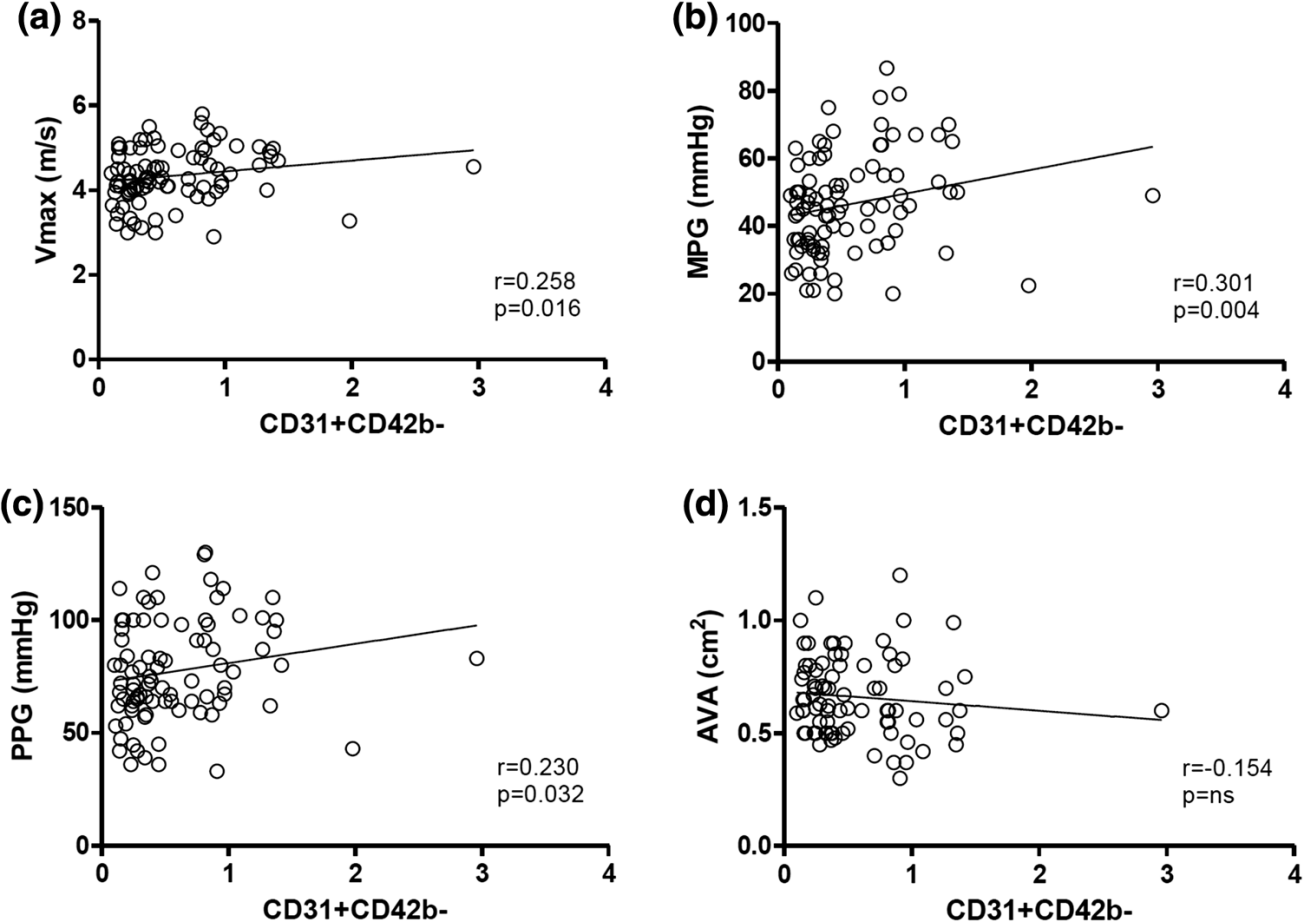
Correlation of CD31+CD42b− EMP levels with parameters of aortic valve function. CD31+CD42b− EMP evidenced a significant correlation with *V*_max_, MPG and PPG, however, no correlation was found with aortic valve area (AVA)

Plasma samples were obtained at each study visit and were analyzed for EMP and PMP. EMP were defined as CD62E+ and CD31+/CD42− and PMP as CD31+CD42b+. Pre TAVI levels of CD31+CD42b− EMP correlated with *V*_max_ (*r* = 0.258, *p* = 0.016), MPG (0.301, *p* = 0.004) and PPG (0.230, *p* = 0.032), see Fig. 2. No significant correlation was found for CD31+CD42b− EMP with aortic valve area (AVA). A correlation analysis of all EMP and PMP populations with parameters of aortic valve function is shown in supplementary Table 1.

**Fig. 3:**
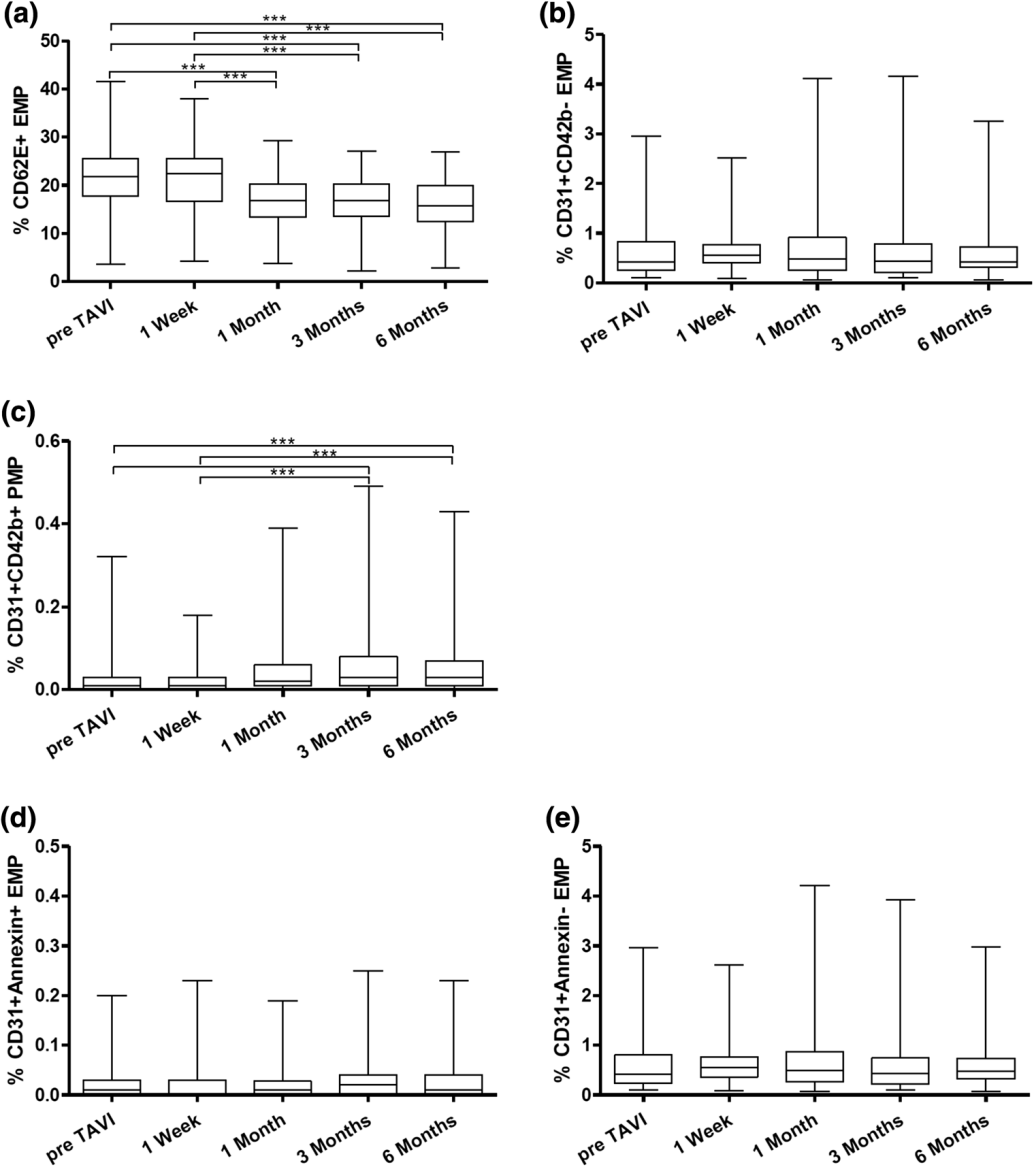
Levels of CD62E decreased significantly after TAVI procedure (**a**). CD31+/CD42b− EMP concentrations did not change significantly after TAVI (**b**). CD31+/CD42b+ PMP evidenced a significant increase during follow-up (**c**). Levels of CD31+/Annexin+ and CD31+/Annexin− EMP were not altered after TAVI (**d**, **e**)

CD62E+ EMP concentration before TAVI was 21.11 % (±6.6 % SD) and declined to 20.99 % (±6.8 % SD) after 1 week, to 16.63 % (±5.4 % SD, *p* < 0.0001) after 1 month, to 17.08 % (±4.6 % SD, *p* < 0.0001) after 3 months and to 15.94 % (±5.4 % SD, *p* < 0.0001) after 6 months (see Fig. 3). No statistically significant differences were found for CD31+/CD42− EMP: pre TAVI 0.57 % (±0.45 % SD), 1 week 0.65 % (±0.45 % SD), 1 month 0.66 % (±0.65 % SD), 3 months 0.69 % (±0.76 % SD) and 6 months 0.62 % (±0.55 % SD).

Interestingly, CD31+/CD42+ PMP tended to increase over time after TAVI procedure. Base line concentration was 0.026 % (±0.0049 % SD), after 1 week 0.024 % (±0.0038 % SD), 1 month 0.052 % (±0.0080 % SD, *p* = 0.0003), 3 months 0.060 % (±0.0101 % SD, *p* < 0.0001) and 6 months 0.66 % (±0.0100 % SD, *p* < 0.0001).

We also analyzed CD31+/Annexin+ and CD31+/Annexin− EMP. Statistically significant differences were found neither for CD31+/Annexin+ nor CD31+/Annexin− EMP. The basic mean level before TAVI for CD31+/Annexin+ EMP was 0.022 % (±0.0041 % SD), after 1 week 0.021 % (±0.0043 % SD), 1 month 0.022 % (±0.0038 % SD), 3 months 0.035 % (±0.0056 % SD) and after 6 months 0.031 % (±0.0049 % SD). Similar results were found for CD31+/Annexin− EMP. Mean concentration before TAVI was 0.56 % (±0.048 % SD), after 1 week 0.64 % (±0.047 % SD), 1 month 0.68 % (±0.071 % SD), 3 months 0.69 % (±0.086 % SD) and after 6 months 0.63 % (±0.056 % SD).

No differences were found between valve types or the approach that was used (transfemoral or transapical). Based on our clinical source data we have performed a comprehensive correlation analysis of EMP and PMP data points and correlated them to clinical features and also complications (using VARC-2 criteria) and valve types. Unfortunately and likely due to the small sample size in sub-groups we have not found any relevant correlations. When performing Bonferroni-Holm correction for multiple testing, no statistically significant correlations were found.

## Discussion

Previous studies have confirmed that elevated levels of microparticles are associated with progression of cardiovascular disease [29]. It was shown that high levels of CD62E+ EMPs are associated with a worse outcome and higher rates of hospitalization in patients with stroke history [30]. Dehl et al. demonstrated in 2008 that EMP and PMP are elevated in the plasma of patients with severe aortic stenosis [31]. More recently, Horn et al. provided evidence that TAVI procedure reduces the number of circulating CD31+/CD42b− and CD62E+ EMP [25].

Here, in this study we sought to investigate the role of CD62E, CD31+/CD42b−, CD31+/Annexin+ and CD31+/Annexin− EMP and CD31+/CD42b+ PMP in patients with severe aortic stenosis undergoing TAVI over a longer follow-up period of 6 months. Regarding functional parameters of aortic stenosis, we found a significant correlation of CD31+/CD41b− but not CD62E+ EMP with *V*_max_, MPG and PPG. Interestingly, no correlation was found for EMP levels with aortic valve area. This could be related to the fact the parameters for *V*_max_, MPG and PPG evidenced a greater statistical spread compared to AVA. Due the definition of aortic stenosis, AVA values are within a rather narrow range (between approximately 0.5 and 1.0 cm^2^). It is known that EMP are released upon shear stress from activated endothelial cells [20, 32]. In patients with severe aortic stenosis, vascular shear stress is constantly elevated leading to an increased expression of adhesion molecule expression [33, 34]. It was speculated that this pathophysiologic process could lead to further progression of valve calcification and dysfunction.

The main focus of our study was to evaluate whether TAVI procedure could lead to alteration or possible decrease in circulating levels of microparticles epitomizing an improvement in vascular integrity and function. We observed that after TAVI a significant decline in CD62E+ EMP occurs as evidenced at follow-up visits at 1, 3 and 6 months (Fig. 2a). Horn et al. already have verified that TAVI can reduce systemic levels of EMP. It was possible to corroborate these data in a larger cohort with a longer follow-up and show that CD62E+ levels remained low over the whole follow-up period of 6 months. In contrast to the results by Horn et al., however, plasma concentration of CD31+/CD42b− EMP remained at baseline level at every visit after TAVI procedure (Fig. 2b).

Of special interest was the finding that PMP staining positive for CD31+/CD42b+ increased significantly over time after TAVI (Fig. 2c). In the study conducted by Horn et al. no changes in CD31+/CD42b+ PMP levels were recorded after TAVI procedure. In an experimental study by Franca et al. it was shown that PMP levels were not affected by the administration of clopidogrel over a surveillance period of 3 weeks [35]. We can only hypothesize that possible surface interaction effects of platelets and the newly implanted valve could have influenced the generation of PMP. In addition, CD31+/CD42b+ PMP levels might also be a surrogate parameter for beginning thrombus formation on cusps of the newly implanted valve [36, 37]. However, this remains truly speculative and further studies would be warranted to further elucidate the mechanism behind.

In the progression of cardiovascular disease and increased endothelial dysfunction and damage, apoptosis can be triggered leading to the release of CD31+/Annexin+ EMP. The group of Sinning et al. demonstrated that heightened levels of CD31+/Annexin+ EMP are related to significantly increase cardiovascular mortality, need for revascularization and major adverse cardiovascular events [38]. In our study, levels of CD31+/Annexin+ EMP did not evidence any differences during follow-up. One could speculate that TAVI leads to a halt in the progression of endothelial stress and dysfunction as CD31+/Annexin+ did not increase and CD62E+ EMP decreased over time. However, further prospective studies with longer follow-up are needed to provide evidence for this hypothesis.

As the shedding of microparticles is also associated with systemic inflammation and endothelial activation, a vicious circle of hemodynamic shear stress and endothelial dysfunction is present in the pathophysiological setting of AS. It was shown that inflammation might play a fundamental part in the progression of degenerative AS. Microparticles also seem to play a role as signaling factors within the vascular compartment mediating inflammatory, angiogenesis and coagulation [39, 40]. TAVI proved to be a very promising procedure in considerably improving valvular function in patients with severe AS. As levels of CD62E+ EMP were significantly reduced and other fractions of EMP did not increase during follow-up, one could argue that one factor for the progression of vascular dysfunction was taken out of the equation, thus leading to an improvement in endothelial function.

## Conclusion

In conclusion, TAVI led not only to a procedure-related improvement in hemodynamic parameters, also endothelial dysfunction may have been alleviated by the procedure. The reduction in transvalvular gradients and less hemodynamic shear stress seems also to have possible beneficial effects on endothelial homeostasis.

## Electronic supplementary material

Below is the link to the electronic supplementary material.

Supplementary Table 1: Additional correlation of EMP and PMP levels with parameters of aortic valve function in echocardiography (XLS 28 kb)
